# Adult case of partial trisomy 9q

**DOI:** 10.1186/1471-2350-11-26

**Published:** 2010-02-16

**Authors:** Keith Tiong, Andrew Cotterill, Henrik Falhammar

**Affiliations:** 1 School of Medicine, James Cook University, Queensland, Australia; 2 Department of Paediatric Endocrinology, Mater Children's Hospital, Brisbane, Queensland, Australia; 3 Department of Molecular Medicine and Surgery, Karolinska Institute, Stockholm, Sweden; 4 Department of Endocrinology, Metabolism and Diabetes, Karolinska University Hospital, Stockholm, Sweden

## Abstract

**Background:**

Complete and partial trisomy 9 is the fourth most common chromosomal disorder. It is also associated with various congenital characteristics affecting the cranio-facial, skeletal, central nervous, gastrointestinal, cardiac and renal systems. Very few cases have been reported in adults. Partial trisomy 9q is also associated with short stature, poor growth and growth hormone deficiency. This is the first reported case of an extensive endocrinology investigation of short stature in trisomy 9q and the outcome of growth hormone treatment.

**Case Presentation:**

The case involves a 23-year-old female of pure partial trisomy 9q. The case involves a 23-year old female with pure partial trisomy 9q involving a duplication of 9q22.1 to q32, de novo, confirmed by genetic studies using fluorescene in situ hybridization (FISH) method. The diagnosis was at 6 years of age. She did not demonstrate all the congenital morphologies identified with trisomy 9q disorders especially in relation to multi-organ morphologies. There is also a degree of associated intellectual impairment. At prepuberty, she was referred for poor growth and was diagnosed with partial growth hormone deficiency. She responded very well to treatment with growth hormone and is currently living an independent life with some support.

**Conclusions:**

Trisomy 9q is associated with short stature and failure to thrive. Growth hormone deficiency should be identified in cases of trisomy 9q and treatment offered. This is the first reported case of response to growth hormone replacement in partial trisomy 9.

## Background

Complete and partial trisomy 9 is the fourth most common chromosomal disorder with over 150 reported cases in the literature [1]. It is associated with cranio-facial abnormalities, failure to thrive, pyloric stenosis, gastro-oesophageal reflux and various defects in cardiovascular, central nervous, gastrointestinal and renal systems. Growth hormone deficiency has been described with complete and partial trisomy 9. The complete form of trisomy 9 is associated with early death while very few cases of partial trisomy 9 has been reported in young adults. We present a case of partial trisomy 9q in an adult patient which is, to the best of our knowledge, the first reported case in two aspects: a novel mutation and her response to growth hormone treatment.

## Case Presentation

The female patient was delivered following an uneventful pregnancy via caesarean section for placenta praevia at 35 weeks gestation with a birth weight of 2.18 kg. The mother, aged 29 years, gave no history of exposure to radiation or mutagens. The immediate family members were healthy and had had normal development. She exhibited dysmorphic features: broad forehead, circular ears, widely spaced nipples, hypertelorism and deep set eyes. The child was noted to be developing slowly from 6 months, in particular fine and gross motor skills. She walked at the age of 30 months. Psychological assessments led to her receiving education support for learning difficulties, labile emotions and anxiety whilst attending main stream schooling. At 4 years she was growing poorly (Ht SDS -4.0, Wt SDS -4.0).

The child was fully investigated at 6 years. Genetic testing revealed additional genetic material of indeterminate source at chromosome 9q 22. The parents had normal chromosomal pattern. Pituitary function studies (including insulin tolerance test) revealed partial GH deficiency (peak GH level 15.7 U/l) whilst gonadotrophin, cortisol, and thyroid studies were normal. Renal ultrasound was normal.

**Figure 1:**
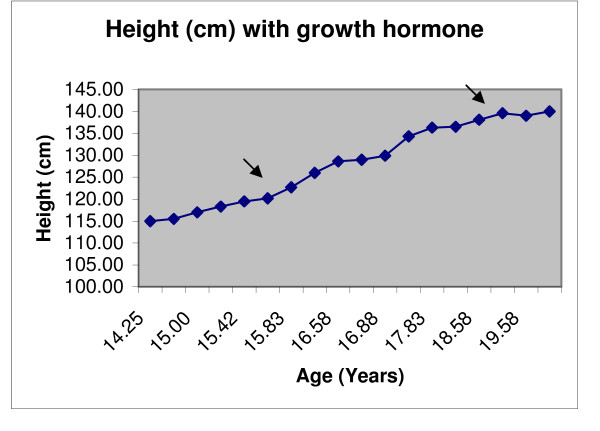
**Height with growth hormone treatment**. Growth Hormone treatment started at 15 years 8 months and stopped at 19 years 2 months.

At 14 years she was prepubertal and had reached a height of 115 cm (SDS -3.0) and weight of 23kgs (SDS -2.0). Biochemical and clinical investigations were repeated (Table 1 and 2). Glucose tolerance was normal. Sleep and exercise GH studies supported the diagnosis of GH deficiency (though these studies were performed without sex steroid priming). Bone age was 11.0 years (Greulich and Pyle) with a delay of 3 years. GnRH stimulation test suggested a pre-pubertal response (LH peak, 3.4 U/l [normal pubertal peak >5.0]; FSH peak, 9.9 U/l [1.0-8.0]) as did pelvic ultrasound showing prepubertal uterus and ovaries. GH therapy was commenced at 15.6 years on the basis of extreme short stature, slow growth and partial GH deficiency. GH therapy was administered for 3 years, initially 3 mg/wk and increased to 8.6 mg/wk. There was an excellent response to GH therapy; IGF-I normalized and a further growth of 23 cm (Figure 1)(Table 1). At the age of 16 years, she entered puberty and achieved menarche at 18 years, with subsequent regular menstrual cycle.

**Table 1:** Biochemical analysis of a female with partial trisomy 9q treated with growth hormone

**Age (years**)	15	18	23	Ref range
Morning Growth hormone (mU/l)	< 0.5		< 0.5	

Post exercise peak growth hormone (mU/l)	0.7			>10

Sleep test growth hormone (mU/l)	0.8			>10

Insulin like growth factor I (nmol/l)	8.0	57	16	20-80

Insulin growth factor BP3 (mg/l)	1.0	140		1.6-4.0

Follicle stimulating hormone (U/l)	2.0	4.0	2.0	1.0-8.0

Luteinizing hormone (U/l)	< 0.5	11	38	1.0-12.0

Oestradiol (pmol/l)	37	287	369	Prepubertal: <100 Postpubertal: >200

Prolactin (mU/l)	255			71-556

Thyroid stimulating hormone (mU/l)	1.79		2.2	0.30-5.00

Free thyroxine (pmol/l)	14.1	11.0	10.0-25.0	

Morning cortisol (nmol/l)	288	389	200-700	

Fasting glucose (mmol/l)	4.5		4.0-6.0	

Oral glucose tolerance test 2 h value (mmol/l)	7.6		<8.9	

**Table 2:** Clinical staging at 15 and 18 years of age

Tanner staging	Age 15 yrs	Age 18 yrs
Breast development (stage)	1	3

Pubic hair growth (stage)	1	3

Bone age (years)	11	14 (mature)

**Figure 2:**
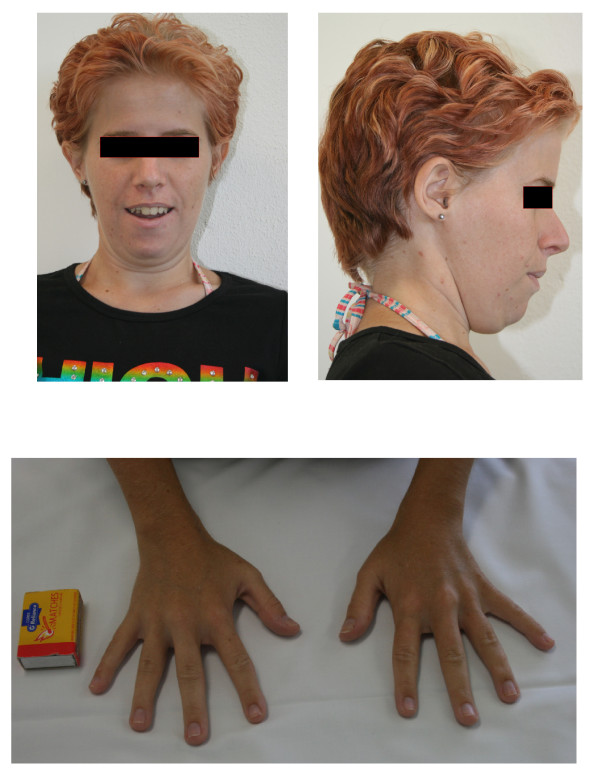
**Adult female with trisomy 9q illustrating the facial features and the hands**.

**Figure 3:**
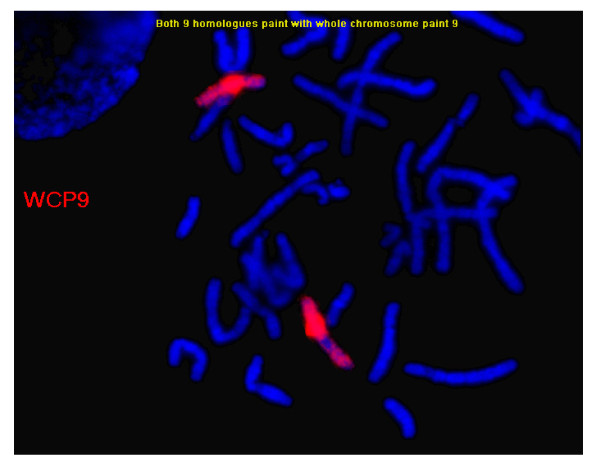
**FISH analysis with whole chromosome 9 paint and *ABL gene *specific probes (band *9q34.1*)**.

**Figure 4:**
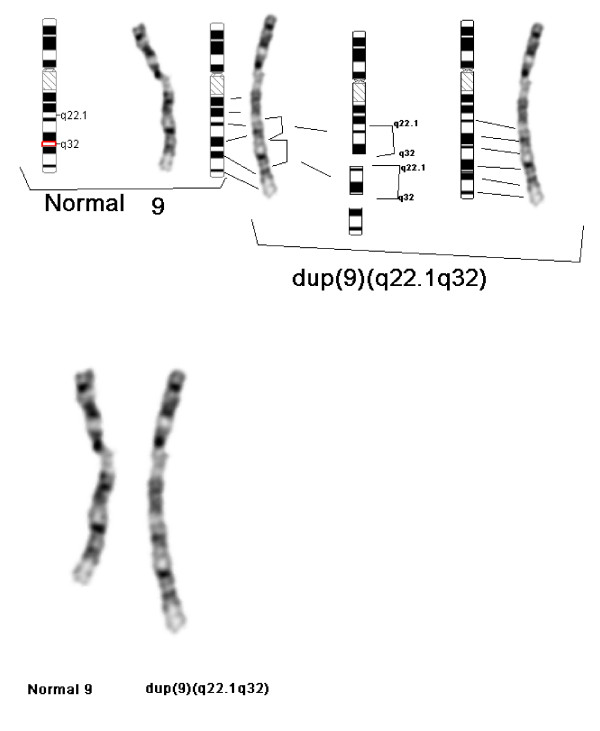
**Idiogram and partial karyotype revealing duplication of *9q***.

At 23 years of age, the patient was re-evaluated. She had intellectual impairment and some obsessive compulsive behaviour. Further dysmorphic features were apparent: broad forehead, short neck, low set ears (Figure 2) and wide gap between the first and second toes. She had nasal speech, perhaps secondary to velo-pharyngeal insufficiency. Echocardiogram and renal ultrasound were normal. A MRI of brain was normal including the pituitary gland except for an abnormal appearance of the corpus callosum with thickening of genu and rostrum. Endocrine investigations were normal apart from low IGF-I level (Table 1). Repeat chromosome analysis with detailed band analysis with Vysis probe and fluorescene in sit hybridization (FISH) with subtelomere probe and ABL specific probe on 9q34 (Abbott-Vysis, Downer's Grove, Il) revealed a pure partial trisomy of chromosome 9 from band 9q22.1 to 9q32 with the additional genetic material considered a duplication of the long arm of chromosome 9 (Figure 3 &4).

## Conclusions

Complete trisomy 9 is associated with spontaneous abortion and those surviving pregnancy rarely live beyond childhood. Subjects with partial trisomy 9 seem to have better prognosis with few reported cases of reaching adulthood [2]. The aetiology and risk factors for this condition are not known [2]. Suspicion of complete and partial trisomy 9 can be identified and confirmed by chorionic venous sampling in the first trimester or later by amniocentesis [3].

The chromosome studies in our patient indicated breakpoints very similar to a previous report of an infant with trisomy 9q and multiple congenital abnormalities together with GH deficiency [4]. Our case did not demonstrate all of the previously reported abnormalities seen in other cases of trisomy 9q [4-6]. As with our case, impaired psychomotor development is common in trisomy 9q, although normal psychomotor development has been described (Table 3) [7]. The MRI demonstrated features of corpus callosum dysgenesis and it has been suggested that all individuals with different kinds of trisomy 9 should be screened with a MRI brain because of the high risk of structural disorder affecting the central nervous system with this disorder [1].

**Table 3:** Clinical findings of reported cases of pure partial trisomy *9q *with duplication (*q22.1*→) [4,11-13]

References	1	2	3	4	5
Age	23 years	1 year	17 years	40 years	7 months

Reduced birth weight	+	+			

Failure to thrive	+	+	+		+

Learning disability	+	+	+	+	

Microcephaly		+	+		

Hypertelorism	+				+

Other facial morphologies	+	+			

High arched palate				+	

Short neck	+				

Cardiac abnomalies			+		

CNS abnomalies		+		+	

Pyloric stenosis		+		+	

Digital abnomalies	+	+		+	

Growth hormone deficiency	+	+			

*Hypothyroidism			+		

*Oesophageal atresia			+		

Comparison of trisomic sections of partial trisomy 9q

(1) Present study. (2009) 46, XY, dup(9)(q22.1→32) de novo

(2) Heller, A et al. (2000) 46, XX, dup(9)(pter→q22.1::q31.1→q22.1::q31.1

q22.1::q31.1→qter)

(3) Hengstschläger, M et al. (2004) 46, XX, dup(9)(q22.1→q33) de novo

(4) Maraschio et al. (1998) 46, XY, dup(9)(q22.1→q31.3)

(5) Narahara et al. (1986) dup(9)(q22.1→q31.3)

As a patient with partial trisomy 9, she survives into adulthood and as a consequence she has multiple physical and psychological challenges. The degree of deformities in trisomy 9 is associated with the degree of trisomic cell expression in various organs. Our patient had relatively minimal dysmorphic features which could be consistent with the degree of duplication of external chromosome 9 [7]. This is the first case demonstrating the benefits of GH therapy. Trisomy 9q has been shown to have GH deficiency (Table 3) but have not been treated with GH, and although our patient only had partial GH deficiency, she responded very well. The genes for GH and chorionic somatomammotropin have been mapped to chromosome 17 [8] and not chromosome 9. However, the *LHX 3 gene*, responsible for a rare syndrome with combined pituitary hormone deficiency and pathological pituitary anatomy, is located at 9q34 [9]. Our patient did not demonstrate any other pituitary deficiency or pituitary defects on MRI, hence, the exact cause of the GH deficiency is unclear. Growth retardation is common in chromosome disorders, and is often attribute to the aneuploidy [10]. One example of such chromosome disorder with some similarities to partial trisomy 9q is partial trisomy 1q [10]. Since growth retardation is common in chromosome disorders, we suggest all patients with partial trisomy 9 to be evaluated for GH deficiency and GH treatment initiated generously.

## Abbreviations

(Ht): Height; (Wt): weight; (SDS): standard deviation scores; (GH): growth hormone; (GnRH): gonadotropin releasing hormone; (LH): luteinising hormone; (FSH): follicle stimulating hormone; (IGF): insulin growth factor; (MRI): magnetic resonance imaging; (LHX): LIM-homeodomain gene.

## Consent

Written consent was obtained from subject and her mother for the publication of this case report and accompanying images. A copy of written consent is available for review.

## Competing interests

The authors declare that they have no competing interests.

## Authors' contributions

HF managed the patients during adulthood, conceived the idea for the article and contributed to the development and final draft of the manuscript. KT was responsible for the research, data gathering and development of the manuscript. AC managed the patient during childhood, provided specialised paediatric endocrinology opinion and contributed to the final draft of the manuscript. All the authors read and approved the final manuscript.

## Pre-publication history

The pre-publication history for this paper can be accessed here:

http://www.biomedcentral.com/1471-2350/11/26/prepub
